# Supplementary material for: Growth of non-layered 2D transition metal nitrides enabled by transient chloride templates
Source: Nat Commun. 2026 Jan 14;17:1615. doi: 10.1038/s41467-026-68321-7 (PMC12905415; doi:10.1038/s41467-026-68321-7)
Supplement: Supplementary file 1 — Supplementary Information [file 41467_2026_68321_MOESM1_ESM.pdf]

Supplementary Information for

Growth of non-layered 2D transition metal nitrides enabled by transient chloride templates

Liqiong He[1,2], Jingwei Wang[1,2,3], Zhengyang Cai[4], Ruiting Liu[1,2], Shengnan Li[1,2], Yunhao Zhang[1,2], Zhi-Yuan Zhang[1,2], Jiarong Liu[1,2], and Bilu Liu[1,2]*

[1]Shenzhen Geim Graphene Center, Shenzhen Key Laboratory of Advanced Layered Materials for Value-added Applications, Institute of Materials Research, Tsinghua Shenzhen International Graduate School, Tsinghua University, Shenzhen 518055, PR China

[2]Key Laboratory of Electrocatalytic Materials and Green Hydrogen Technology of Guangdong Higher Education Institutes, Tsinghua Shenzhen International Graduate School, Tsinghua University, Shenzhen 518055, PR China

[3]School of Flexible Electronics, Sun Yat-sen University, Shenzhen, 518107, PR China

[4]School of Integrated Circuits, Jiangnan University, Wuxi, Jiangsu 214122, PR China

Corresponding authors

Email: bilu.liu@sz.tsinghua.edu.cn (BL)

The growth details of each 2D TMNs and their alloys

(1) 2D VN flakes were synthesized by the reverse-thermal-field (RTF) method (Supplementary Figure 5). VCl_3 powder (1 mg, 98%, Sigma-Aldrich) was placed in a quartz boat located at Field I. The mica substrate was placed above the quartz boat located at Field II. The deposition temperatures of step 1 were 600 °C (Field I) and 100 °C (Field II). During step 2, a mixture gas flow of NH_3 (10 sccm) and H_2 (5 sccm) was introduced, and the conversion temperatures were 100 °C (Field I) and 600 °C (Field II).

(2) The growth process of 2D CrN was similar to VN. $CrCl_3$ powder (1 mg, 98%, Sigma-Aldrich) was placed in a quartz boat located at Field I. The mica substrate was placed above the quartz boat located at Field II. The deposition temperatures of step 1 were 700 °C (Field I) and 100 °C (Field II). During step 2, a mixture gas flow of NH_3 (10 sccm) and H_2 (5 sccm) was introduced, and the conversion temperatures were 100 °C (Field I) and 700 °C (Field II).

(3) The growth process of 2D MnN was similar to VN. $MnCl_2$ powder (1 mg, 98%, Sigma-Aldrich) was placed in a quartz boat located at Field I. The mica substrate was placed above the quartz boat located at Field II. The deposition temperatures of step 1 were 800 °C (Field I) and 200 °C (Field II). During step 2, a mixture gas flow of NH_3 (10 sccm) and H_2 (5 sccm) was introduced, and the conversion temperatures were 200 °C (Field I) and 800 °C (Field II).

(4) The growth process of 2D Fe_2N was similar to VN. $FeCl_3$ powder (1 mg, 98%, Sigma-Aldrich) was placed in a quartz boat located at Field I. The mica substrate

was placed above the quartz boat located at Field II. The deposition temperatures of step 1 were 650 °C (Field I) and 150 °C (Field II). During step 2, a mixture gas flow of NH_3 (10 sccm) and H_2 (5 sccm) was introduced, and the conversion temperatures were 150 °C (Field I) and 650 °C (Field II).

(5) The growth process of 2D CoN was similar to VN. $CoCl_2$ powder (1 mg, 98%, Sigma-Aldrich) was placed in a quartz boat located at Field I. The mica substrate was placed above the quartz boat located at Field II. The deposition temperatures of step 1 were 650 °C (Field I) and 150 °C (Field II). During step 2, a mixture gas flow of NH_3 (10 sccm) and H_2 (5 sccm) was introduced, and the conversion temperatures were 150 °C (Field I) and 650 °C (Field II).

(6) The growth process of 2D h-NiN was similar to VN. $NiCl_2$ powder (1 mg, 98%, Sigma-Aldrich) was placed in a quartz boat located at Field I. The mica substrate was placed above the quartz boat located at Field II. The deposition temperatures of step 1 were 650 °C (Field I) and 150°C (Field II). During step 2, a mixture gas flow of NH_3 (10 sccm) and H_2 (5 sccm) was introduced, and the conversion temperatures were 150 °C (Field I) and 650 °C (Field II).

(7) The growth process of 2D r-NiN was similar to VN. $NiCl_2$ powder (1 mg, 98%, Sigma-Aldrich) was placed in a quartz boat located at Field I. The mica substrate was placed above the quartz boat located at Field II. The deposition temperatures of step 1 were 800 °C (Field I) and 200 °C (Field II). During step 2, a mixture gas flow of NH_3 (10 sccm) and H_2 (5 sccm) was introduced, and the conversion temperatures were 200 °C (Field I) and 800 °C (Field II).

(8) The growth process of 2D r-Co_xNi_yN was similar to VN. A mixed precursor of $CoCl_2$ powder (0.5 mg, 98%, Sigma-Aldrich) and $NiCl_2$ (0.5 mg, 98%, Sigma-Aldrich) was placed in a quartz boat located at Field I. The mica substrate was placed above the quartz boat located at Field II. The deposition temperatures of step 1 were 780 °C (Field I) and 200 °C (Field II). During step 2, a mixture gas flow of NH_3 (10 sccm) and H_2 (5 sccm) was introduced and the conversion temperatures were 200 °C (Field I) and 780 °C (Field II).

(9) The growth process of 2D h-Co_xNi_yN was similar to VN. A mixed precursor of $CoCl_2$ powder (0.5 mg, 98%, Sigma-Aldrich) and $NiCl_2$ (0.5 mg, 98%, Sigma-Aldrich) was placed in a quartz boat located at Field I. The mica substrate was placed above the quartz boat located at Field II. The deposition temperatures of step 1 were 650 °C (Field I) and 140 °C (Field II). During step 2, a mixture gas flow of NH_3 (10 sccm) and H_2 (5 sccm) was introduced, and the conversion temperatures were 140 °C (Field I) and 650 °C (Field II).

(10) The growth process of 2D Co_xFe_yN was similar to VN. A mixed precursor of $CoCl_2$ powder (0.5 mg, 98%, Sigma-Aldrich) and $FeCl_3$ (0.5 mg, 98%, Sigma-Aldrich) was placed in a quartz boat located at Field I. The mica substrate was placed above the quartz boat located at Field II. The deposition temperatures of step 1 were 650 °C (Field I) and 140 °C (Field II). During the step 2, a mixture gas flow of NH_3 (10 sccm) and H_2 (5 sccm) was introduced and the conversion temperatures were 140 °C (Field I) and 650 °C (Field II).

(11) The growth process of 2D Cr_xFe_yN was similar to VN. A mixed precursor of

$CrCl_3$ powder (0.5 mg, 98%, Sigma-Aldrich) and $FeCl_3$ (0.5 mg, 98%, Sigma-Aldrich) was placed in a quartz boat located at Field I. The mica substrate was placed above the quartz boat located at Field II. The deposition temperatures of step 1 were 650 °C (Field I) and 140 °C (Field II). During the step 2, a mixture gas flow of NH_3 (10 sccm) and H_2 (5 sccm) was introduced and the conversion temperatures were 140 °C (Field I) and 650 °C (Field II).

(12) The growth process of 2D $Co_{0.2}Ni_{0.1}Fe_{0.7}N$ was similar to VN. A mixed precursor of $CoCl_2$ powder (0.3 mg, 98%, Sigma-Aldrich), $NiCl_2$ powder (0.3 mg, 98%, Sigma-Aldrich) and $FeCl_3$ (0.3 mg, 98%, Sigma-Aldrich) was placed in a quartz boat located at Field I. The mica substrate was placed above the quartz boat located at Field II. The deposition temperatures of step 1 were 650 °C (Field I) and 140 °C (Field II). During step 2, a mixture gas flow of NH_3 (10 sccm) and H_2 (5 sccm) was introduced and the conversion temperatures were 140 °C (Field I) and 650 °C (Field II).

(13) The growth process of 2D $Cr_xFe_yCo_zN$ was similar to VN. A mixed precursor of $CrCl_3$ powder (0.3 mg, 98%, Sigma-Aldrich), $FeCl_3$ powder (0.3 mg, 98%, Sigma-Aldrich) and $CoCl_2$ (0.3 mg, 98%, Sigma-Aldrich) was placed in a quartz boat located at Field I. The mica substrate was placed above the quartz boat located at Field II. The deposition temperatures of step 1 were 650 °C (Field I) and 140 °C (Field II). During the step 2, a mixture gas flow of NH_3 (10 sccm) and H_2 (5 sccm) was introduced and the conversion temperatures were 140 °C (Field I) and 650 °C (Field II).

(14) The growth process of 2D $Cr_xFe_yCo_zMn_wN$ was similar to VN. A mixed precursor of $CrCl_3$ powder (0.3 mg, 98%, Sigma-Aldrich), $FeCl_3$ powder (0.3 mg, 98%, Sigma-Aldrich), $CoCl_2$ (0.3 mg, 98%, Sigma-Aldrich) and $MnCl_2$ (0.3 mg, 98%, Sigma-Aldrich) was placed in a quartz boat located at Field I. The mica substrate was placed above the quartz boat located at Field II. The deposition temperatures of step 1 were 650 °C (Field I) and 140 °C (Field II). During the step 2, a mixture gas flow of NH_3 (10 sccm) and H_2 (5 sccm) was introduced and the conversion temperatures were 140 °C (Field I) and 650 °C (Field II).

(15) The growth process of 2D $Cr_xFe_yCo_zNi_wN$ was similar to VN. A mixed precursor of $CrCl_3$ powder (0.3 mg, 98%, Sigma-Aldrich), $FeCl_3$ powder (0.3 mg, 98%, Sigma-Aldrich), $CoCl_2$ (0.3 mg, 98%, Sigma-Aldrich) and $NiCl_2$ (0.3 mg, 98%, Sigma-Aldrich) was placed in a quartz boat located at Field I. The mica substrate was placed above the quartz boat located at Field II. The deposition temperatures of step 1 were 650 °C (Field I) and 140 °C (Field II). During the step 2, a mixture gas flow of NH_3 (10 sccm) and H_2 (5 sccm) was introduced and the conversion temperatures were 140 °C (Field I) and 650 °C (Field II).

Calculations of temperature dependent Gibbs formation energy

The temperature dependent Gibbs formation energy $\Delta G_f(T)$[1] was calculated according to the equation 1.

$$\Delta G_f(T)= \Delta H_f(298K)+G^{\delta}(T)- \sum_{i=1}^{N} \alpha_i G_i(T) \qquad (1)$$

where ΔH_f is the standard state formation enthalpy (parts of the compounds was

obtained by density function theory (DFT) calculations), G^{δ} is small relative to the entropic contribution, N is the number of elements in the material compound, α_i is the stoichiometric weight of element i, and G_i is the chemical potentials of element i.

To calculate the value of above G^{δ}, the SISSO (sure independence screening and sparsifying operator) approach was used, according to equation 2.

$$G^{\delta}_{SISSO}(T)\left[\frac{eV}{atom}\right]=\left(-2.48 * 10^{-4} *In(V)-8.94 * 10^{-5}\ mV^{-1}\right)\cdot T+0.181 *In(T)-0.882 \quad (2)$$

where V is the calculated atomic volume (Å^3 atom^{-1}), m is the reduced atomic mass (amu), and T is the absolute temperature (K).

Based on the above Gibbs energy of various compounds and chemical reaction equation for obtaining CrN, CoN, Fe_2N, MnN, NiN and VN materials, the reaction energetics for a chemical reaction A+B=C+D was calculated by equation 3:

$$\Delta G= \Delta G_f(T, D)+\Delta G_f(T, C)-\Delta G_f(T, B)-\Delta G_f(T,A) \quad (3)$$

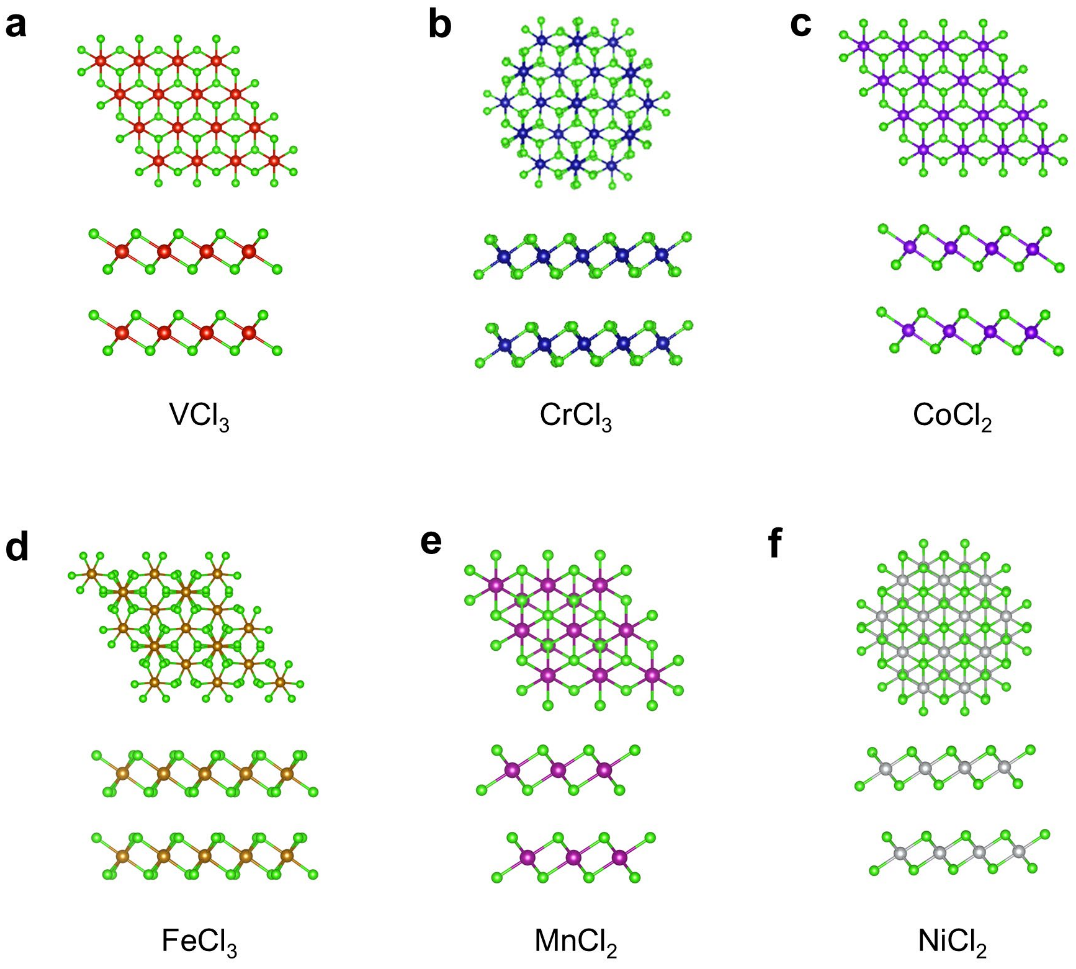

Supplementary Figure 1. Atomic structures of transition metal chlorides. (a)-(f) Top view and side view of (a) VCl_3, (b) $CrCl_3$, (c) $CoCl_2$, (d) $FeCl_3$, (e) $MnCl_2$ and (f) $NiCl_2$.

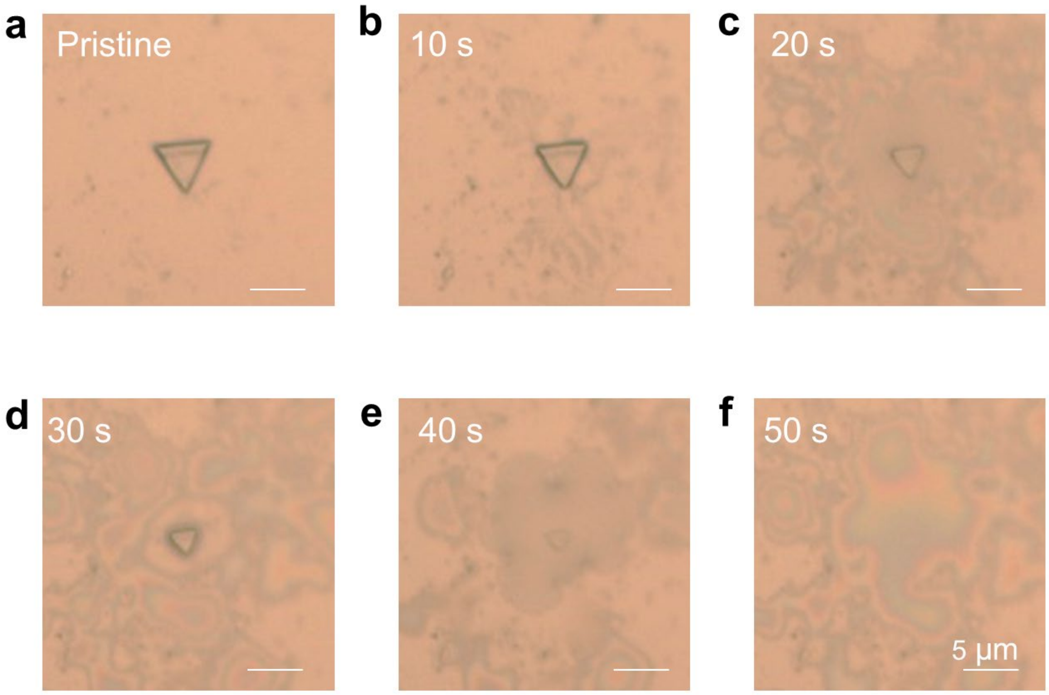

Supplementary Figure 2. Optical microscopy (OM) images of the 2D $CrCl_3$ templates grown on mica substrates. (a) Pristine 2D $CrCl_3$ flakes. (b)-(f) 2D $CrCl_3$ flakes exposed to air for (b) 10 s, (c) 20 s, (d) 30 s, (e) 40 s and (f) 50 s.

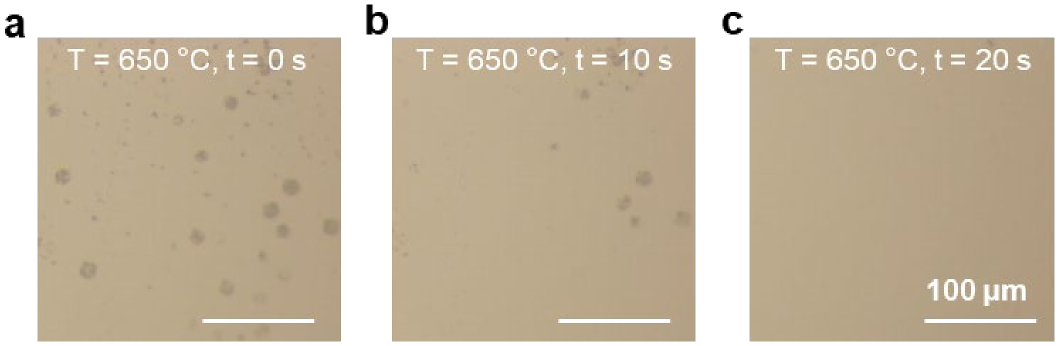

Supplementary Figure 3. Evolution of deposited $CoCl_2$ under high temperature observed by in situ visualization CVD technique. (a) OM image of deposited $CoCl_2$ in Ar atmosphere under 650 °C. (b)-(c) OM images of deposited $CoCl_2$ in Ar atmosphere under 650 °C for (b) 10 s and (c) 20 s.

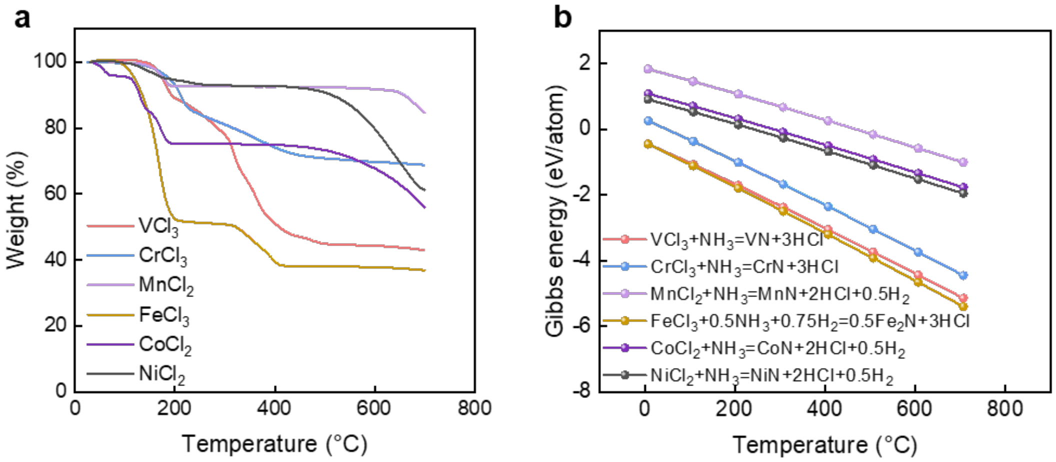

Supplementary Figure 4. Physical and chemical properties of transition metal chlorides. (a) TGA curves of the VCl_3, $CrCl_3$, $CoCl_2$, $FeCl_3$, $MnCl_2$ and $NiCl_2$. (b) The Gibbs energy barrier of the solid-to-solid conversion from transition metal chlorides to their corresponding nitrides at different temperatures.

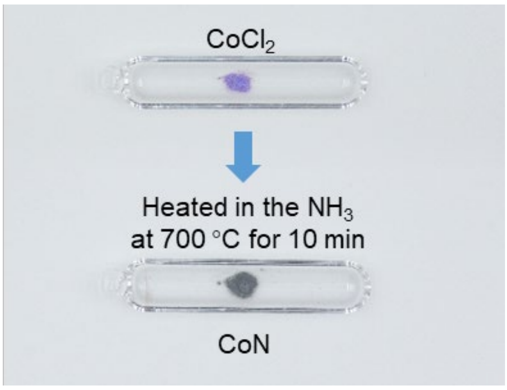

Supplementary Figure 5. Conversion of $CoCl_2$ precursors to CoN when heated in the NH_3 atmosphere at 700 °C for 10 min.

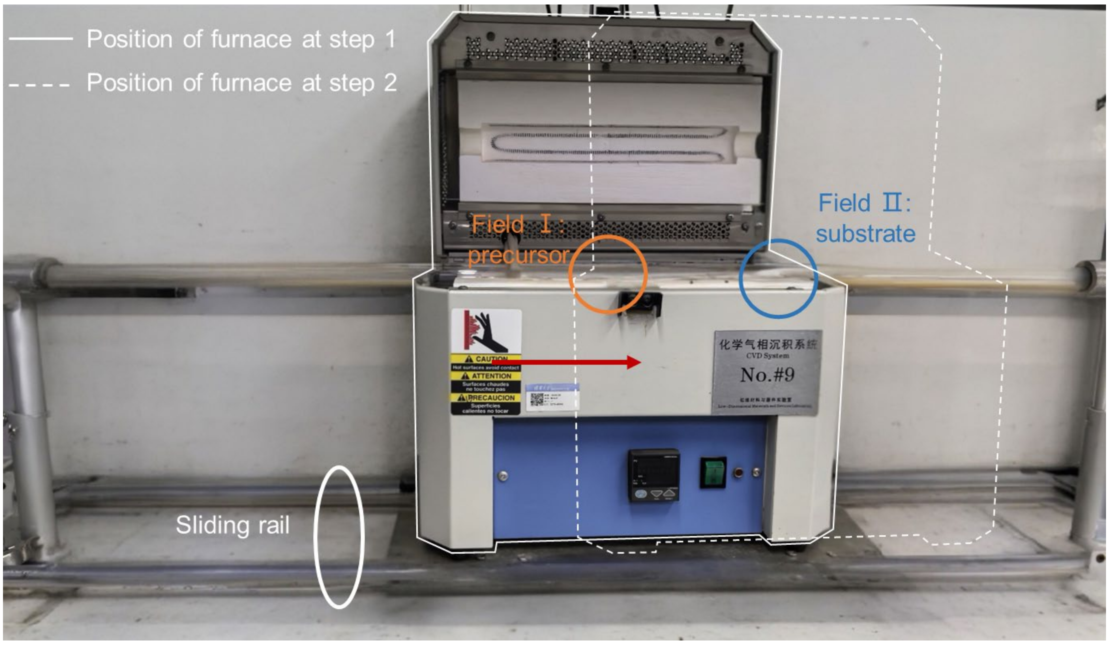

Supplementary Figure 6. Photo of the setup used to achieve RTF process.

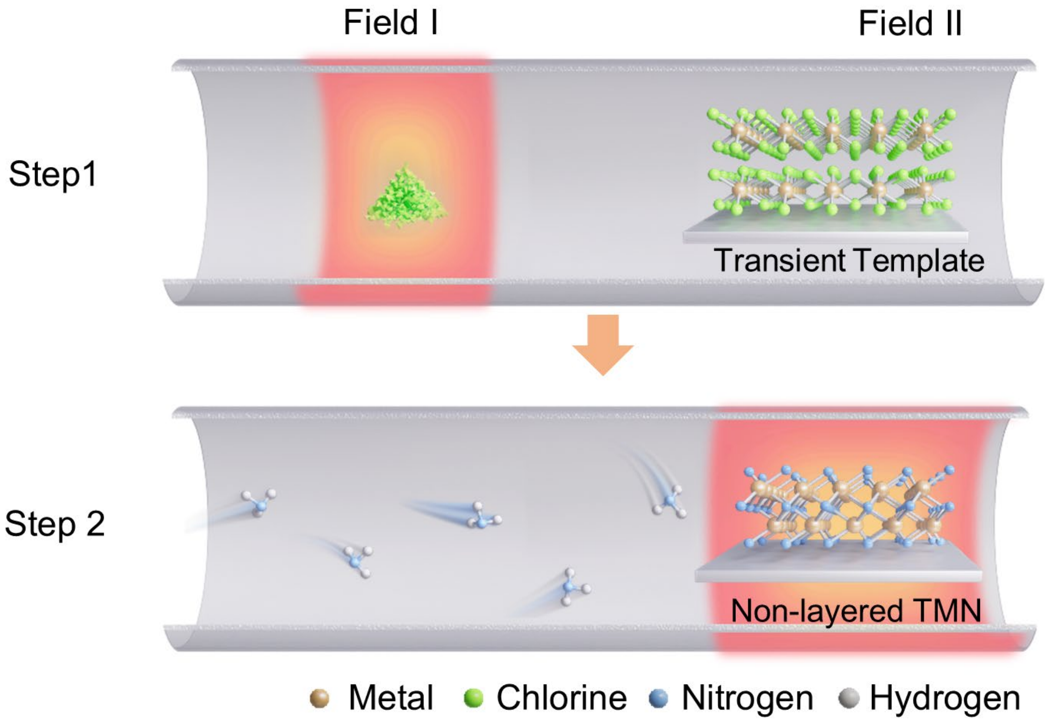

Supplementary Figure 7. Scheme to illustrate the two-step RTF method.

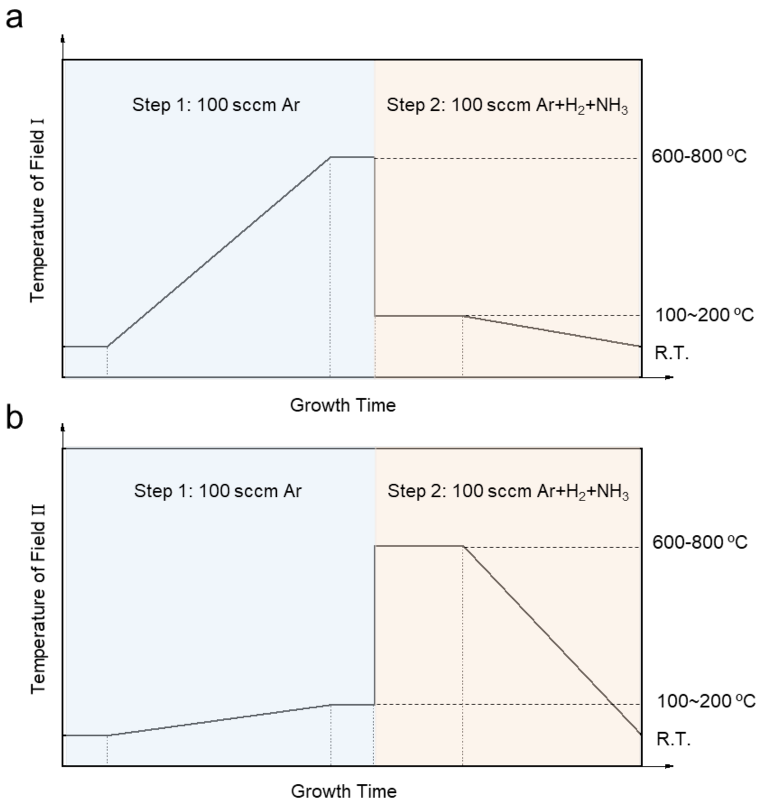

Supplementary Figure 8. **Temperature profile of the growth process of RTF method.** (a) Temperature profile of Field I, where the precursor located. (b) Temperature profile of Field II, where the substrate located.

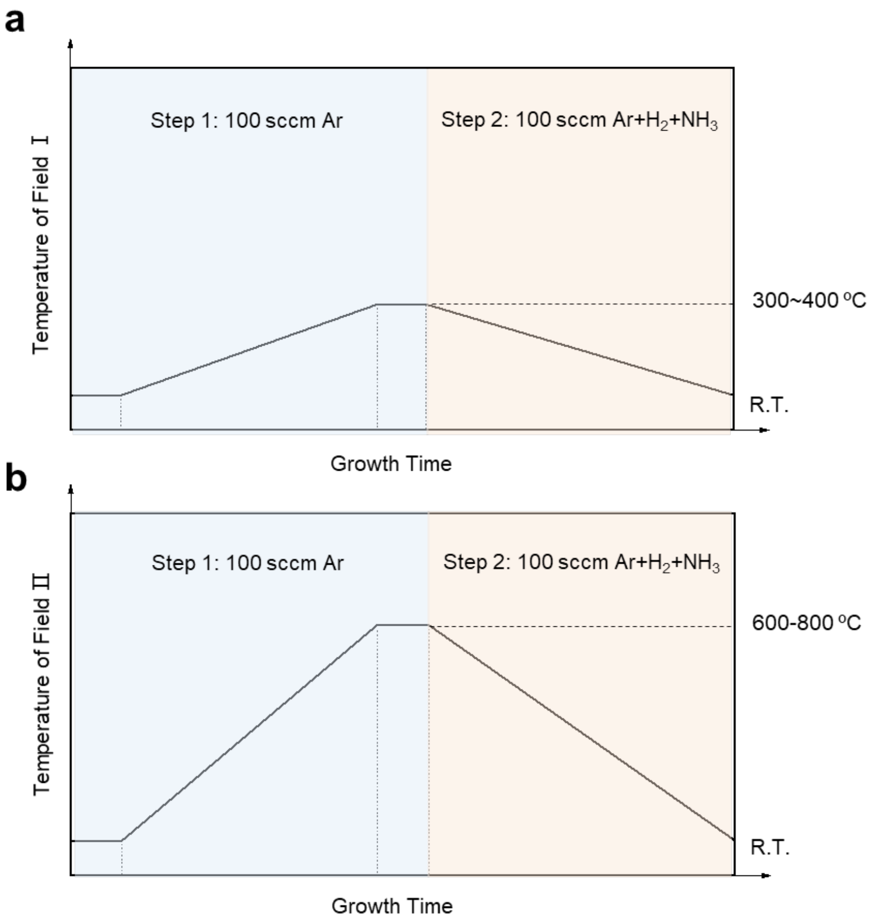

Supplementary Figure 9. Temperature profile of the traditional CVD method. (a) Temperature profile of the precursor during growth, and the precursor is located at Field I. (b) Temperature profile of the substrate during growth, and the substrate is located at Field II.

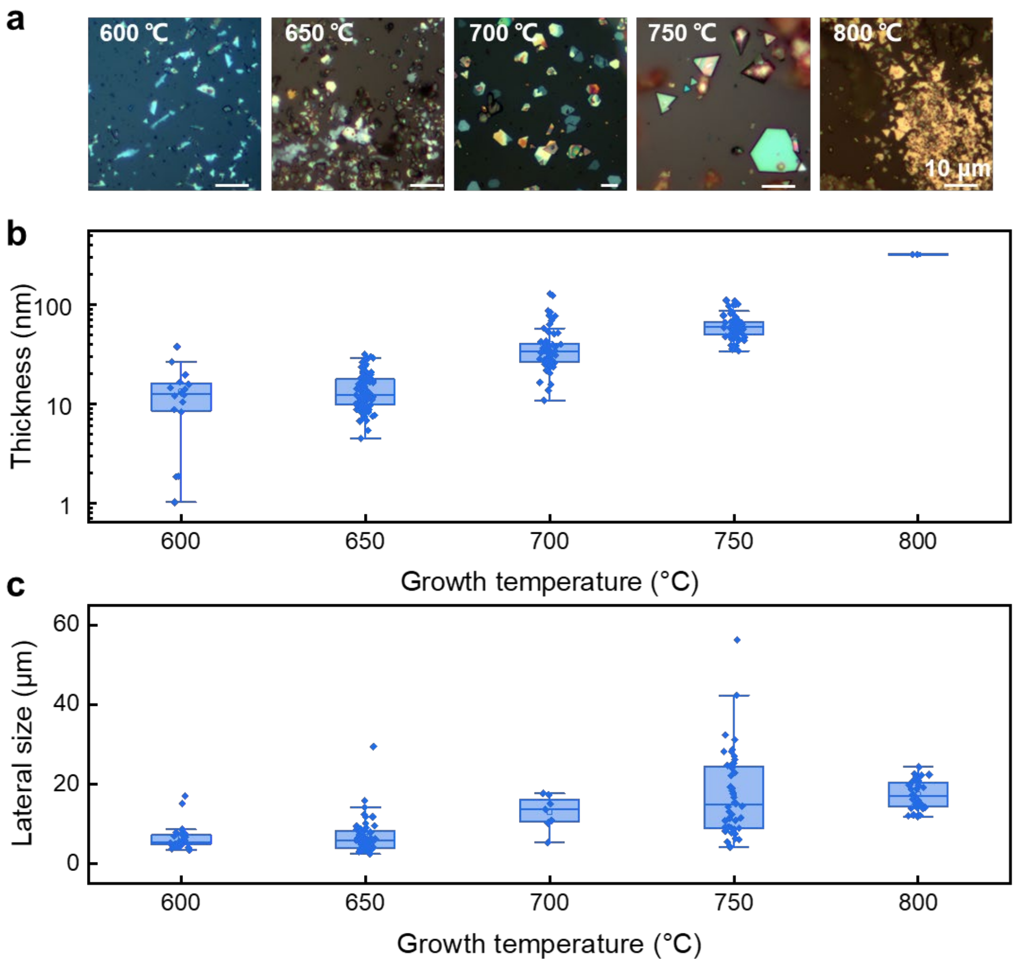

Supplementary Figure 10. The relationship between morphology of CoN and growth temperature. (a) OM images of CoN with increased temperature from 600 °C to 800 °C, and all the growth time were 5 min. (b) The thicknesses of CoN under different growth temperatures, which were obtained by AFM measurements of 249 flakes. The center line, box limits, and whiskers represent the median, quartiles (Q1/Q3), and 1.5×IQR range, respectively. (c) The lateral sizes of CoN under different growth temperature. The lateral size was obtained by OM characterization. Box plots summarize lateral size at each growth temperature. The center line, box limits, and whiskers represent the median, quartiles (Q1/Q3), and 1.5×IQR range, respectively.

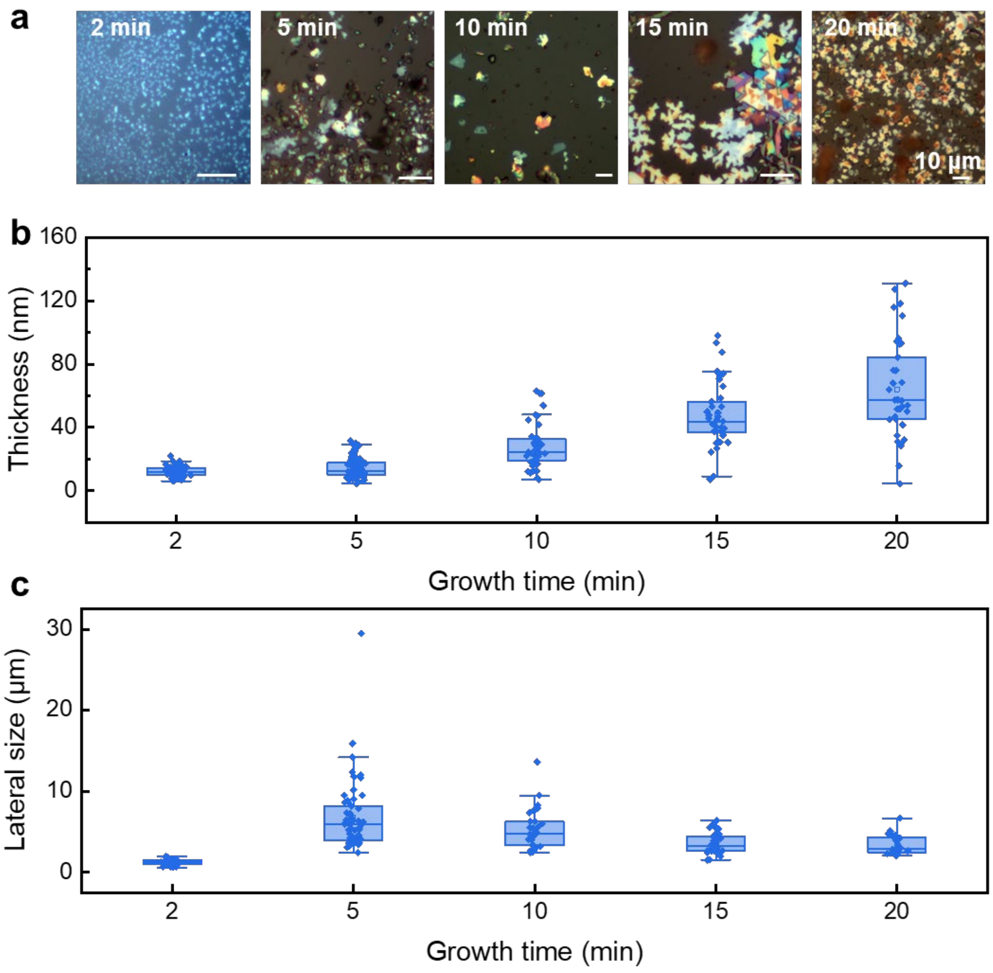

Supplementary Figure 11. The relationship between morphology of CoN and growth time. (a) OM images of CoN with increased growth time from 2 min to 20 min, and all the growth temperatures were 650 °C. (b) The thicknesses of CoN with different growth time, which were obtained by AFM measurements of 293 flakes. The results show that when the growth time are lager, the average thickness will be larger and the range will be wider. The center line, box limits, and whiskers represent the median, quartiles (Q1/Q3), and 1.5×IQR range, respectively. (c) The lateral sizes of CoN with different growth time. The lateral size was obtained by OM characterization. The results shows that when the growth time is 5 min, the average lateral size will be the largest. The center line, box limits, and whiskers represent the median, quartiles (Q1/Q3), and 1.5×IQR range, respectively.

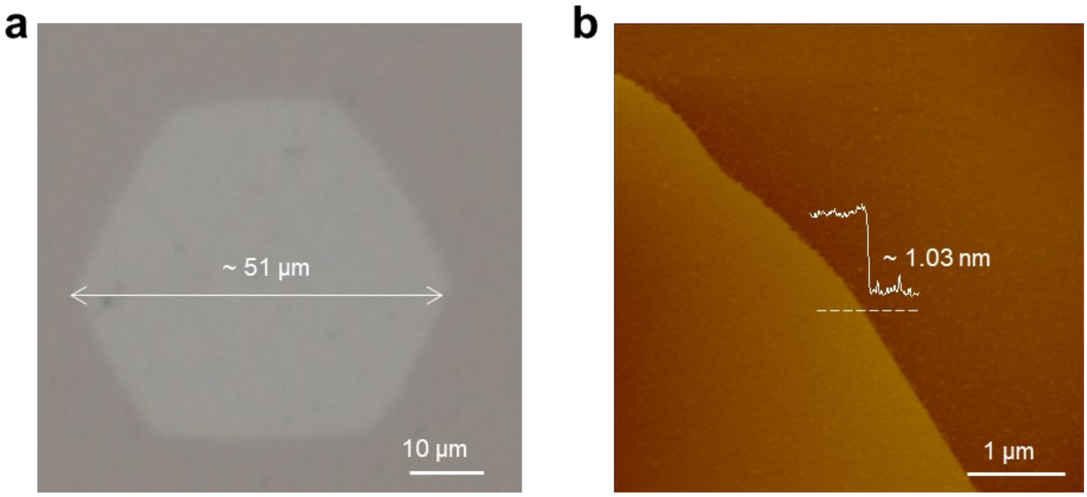

Supplementary Figure 12. OM and AFM image of 2D CoN. (a) The OM image of 2D CoN with the lateral size of 51 μm. (b) The AFM image of the 2D CoN with the thickness of 1.03 nm.

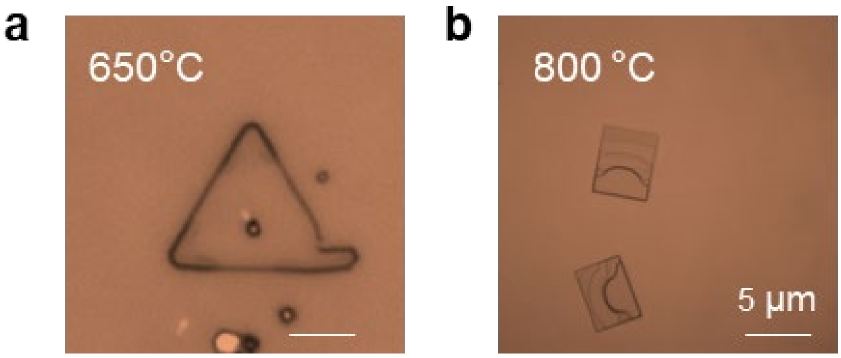

Supplementary Figure 13. OM images of 2D nickel chloride templates obtained at different growth temperatures. (a) OM image of a triangular/hexagonal shape 2D template when the growth temperature is 650 °C. (b) OM image of a rectangular shape 2D template when the growth temperature is 800 °C.

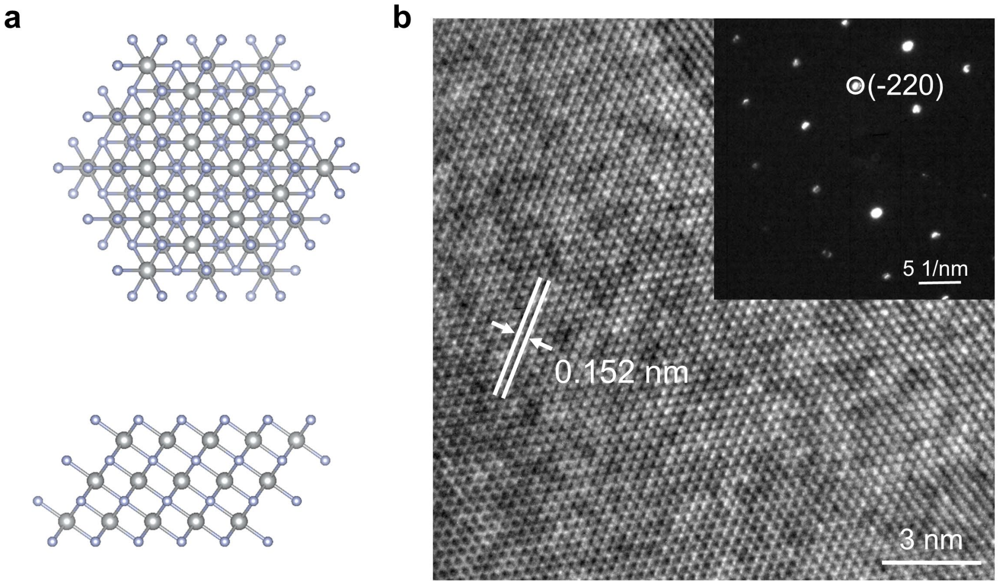

Supplementary Figure 14. Structural characterization of h-NiN. (a) Atomic structure of NiN (PDF#97-016-1755) from the top view and side view. (b) The HRTEM and SAED pattern of h-NiN. The 2D h-NiN has a lattice spacing of 0.152 nm, which is assigned to the (220) plane.

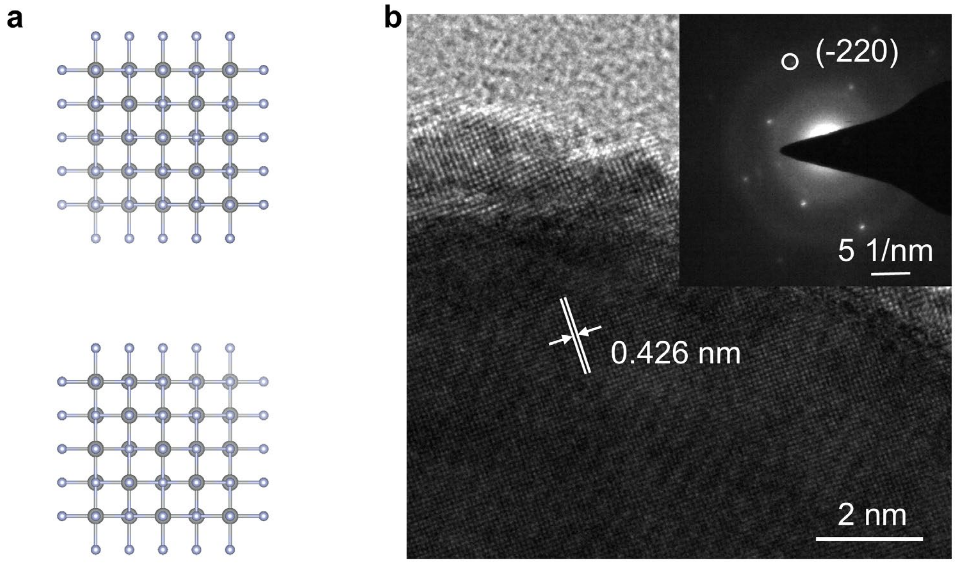

Supplementary Figure 15. Structural characterization of r-NiN. (a) Atomic structure of NiN (PDF#97-016-1755) from the top view and side view. (b) The HRTEM and SAED pattern of t-NiN. The 2D r-NiN has a lattice spacing of 0.426 nm, which is assigned to the (100) plane.

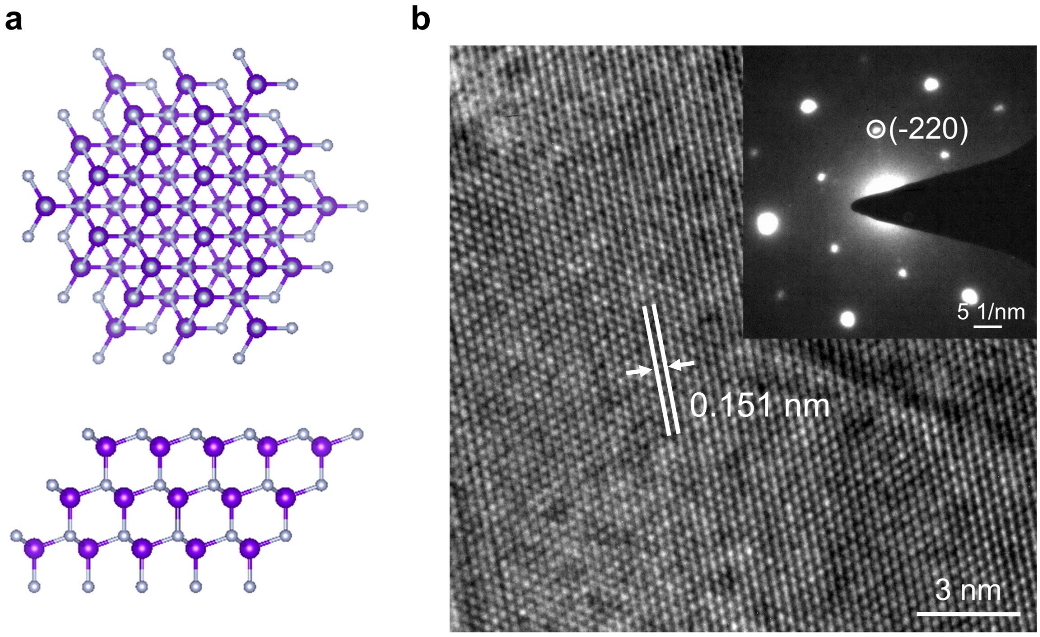

Supplementary Figure 16. Structural characterization of CoN. (a) Atomic structure of CoN (PDF#97-007-9936) from the top view and side view. (b) The HRTEM and SAED pattern of CoN. The 2D CoN has a lattice spacing of 0.151 nm, which is assigned to the (220) plane.

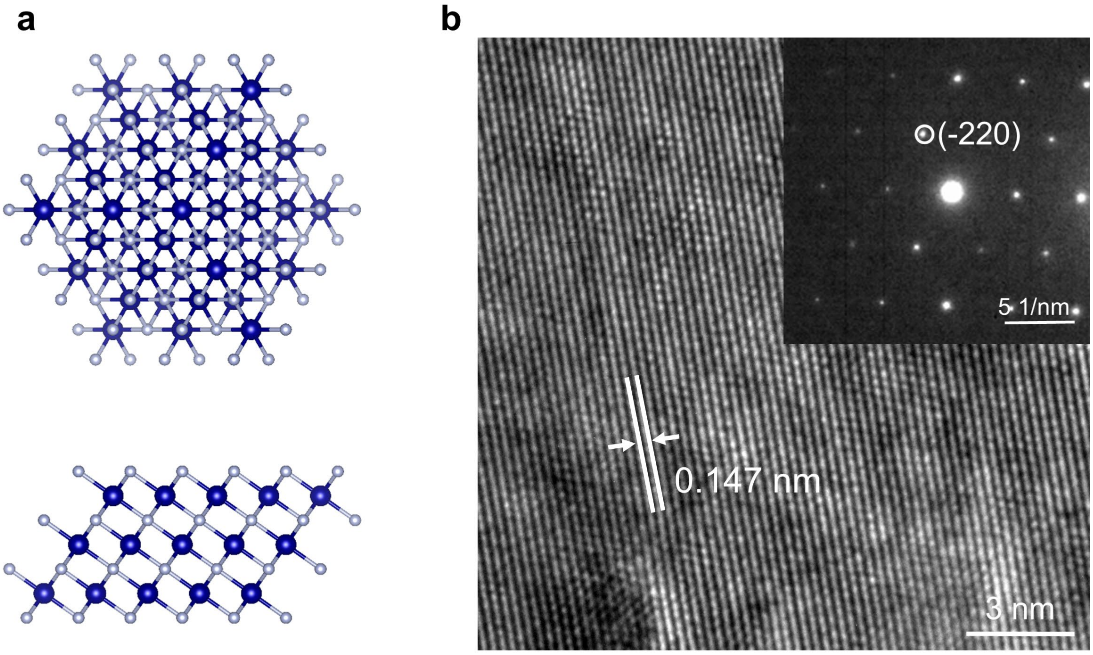

Supplementary Figure 17. Structural characterization of CrN. (a) Atomic structure of CrN (PDF#97-015-2819) from the top view and side view. (b) The HRTEM and SAED pattern of CrN. The 2D CrN has a lattice spacing of 0.147 nm, which is assigned to the (220) plane.

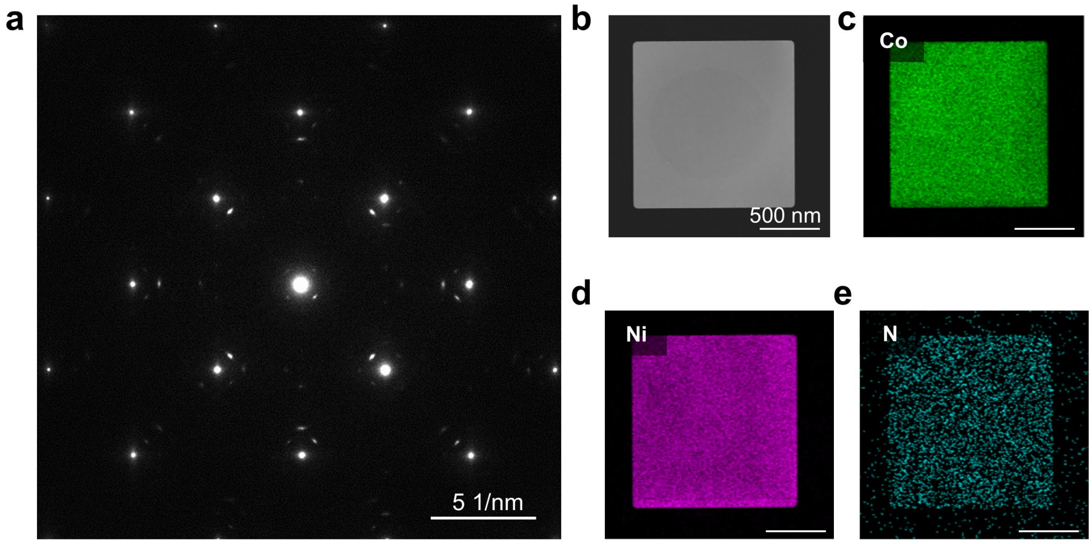

Supplementary Figure 18. Structural and composition characterization of t-Co_xN_iN. (a) SAED patterns of the 2D t-Co_xN_iN flake. (b) Low magnification TEM image of the t-Co_xN_iN. (c)-(e) Elemental EDS mapping of Co, Ni and N atoms.

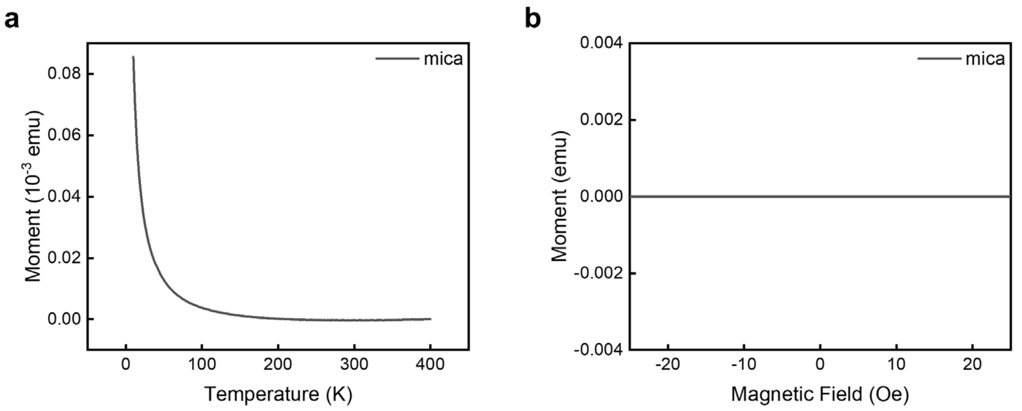

Supplementary Figure 19. Magnetic property of bare mica substrate. (a) M-T curve of mica substrate measured by SQUID. (b) M-H loop of pure mica measured by SQUID at 10 K.

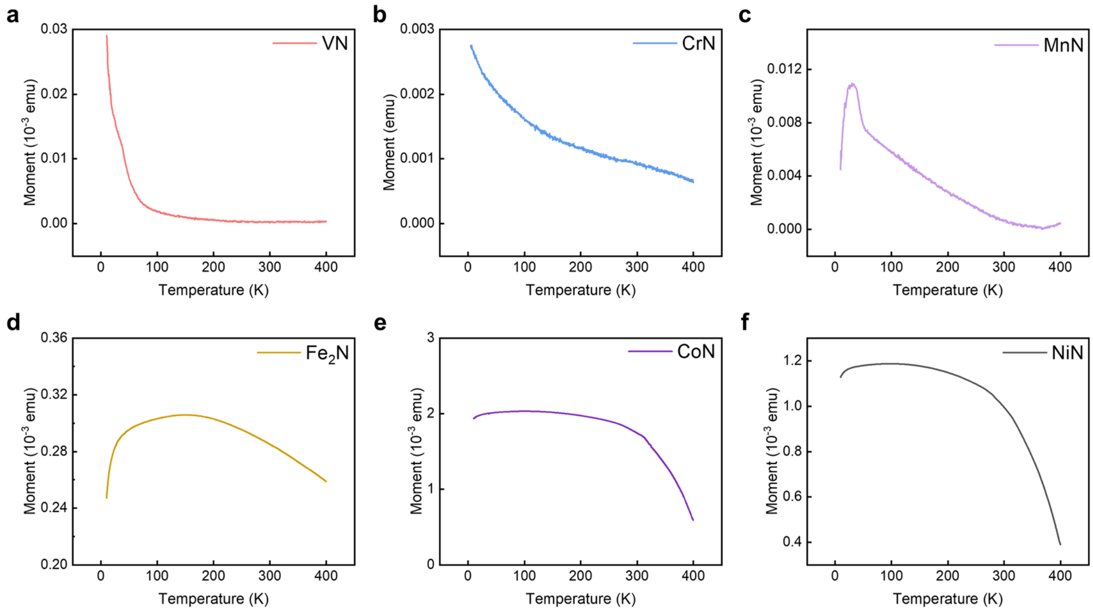

Supplementary Figure 20. Magnetic properties of 2D TMNs. (a)-(f) M-T curves of (a) VN, (b) CrN, (c) MnN, (d) Fe_2N, (e) CoN and (f) NiN measured by SQUID.

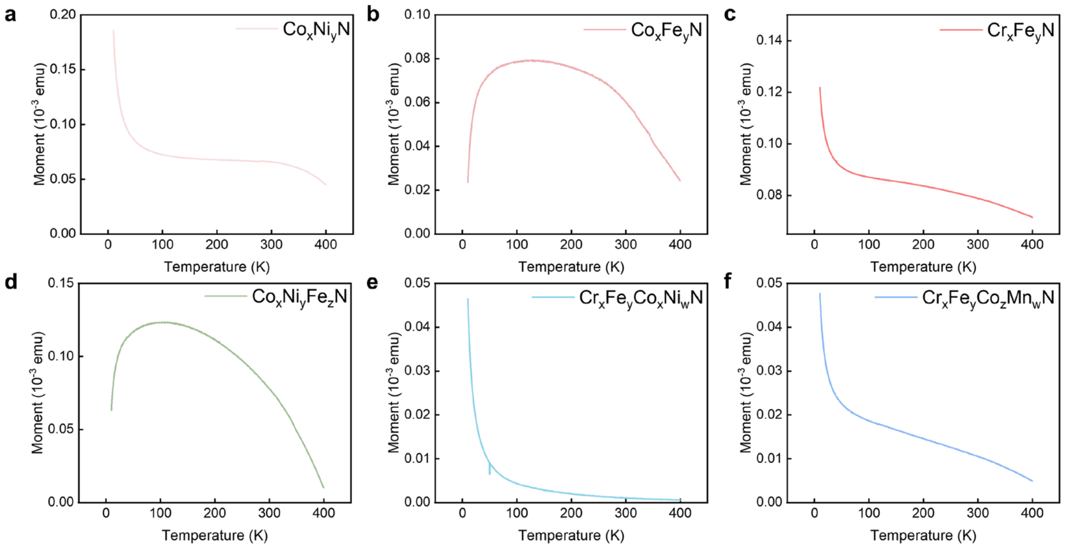

Supplementary Figure 21. Magnetic properties of 2D TMNs. (a)-(f) M-T curves of (a) Co_xNi_yN, (b) Co_xFe_yN, (c) Cr_xFe_yN, (d) $Co_{ox}Ni_yFe_zN$, (e) $Cr_xFe_yCo_zNi_wN$ and (f) $Cr_xFe_yCo_zMn_wN$ measured by SQUID.

Supplementary Table 1. Statistics of M_s and H_C of the 2D TMNs and 2D TMN alloys

2D materials	M_s (emu)	H_C (Oe)
VN	0.044	0
CrN	0.068	188
MnN	0.031	0
Fe_2N	0.377	252
CoN	2.981	405
NiN	1.207	18
Co_xNi_yN	0.099	0
Co_xFe_yN	0.115	250
Cr_xFe_yN	0.208	256
$Co_xNi_yFe_zN$	1.207	759
$Cr_xFe_yCo_zNi_wN$	0.020	0
$Cr_xFe_yCo_zMn_wN$	0.130	0

Supplementary References

1 Bartel, C. J. *et al.* Physical Descriptor for The Gibbs Energy of Inorganic Crystalline Solids and Temperature-Dependent Materials Chemistry. *Nat. Commun.* **9**, 4168 (2018).
